# The coupled study of metal concentrations and electron paramagnetic resonance (EPR) of lichens (*Hypogymnia physodes*) from the Świętokrzyski National Park—environmental implications

**DOI:** 10.1007/s11356-018-2586-x

**Published:** 2018-06-26

**Authors:** Monika Maria Ciężka, Maciej Górka, Magdalena Modelska, Rafał Tyszka, Aleksandra Samecka-Cymerman, Agnieszka Lewińska, Anna Łubek, David Widory

**Affiliations:** 1 Institute of Geological Sciences, Wrocław University, Max Born Sq. 9, 50-204 Wroclaw, Poland; 2 Institute of Soil Sciences and Environmental Protection, Wroclaw University of Environmental and Life Sciences, Grunwaldzka 53, 50-357 Wroclaw, Poland; 3 Department of Ecology, Biogeochemistry and Environmental Protection, Wrocław University, Kanonia St. 6/8, 50-328 Wroclaw, Poland; 4 Faculty of Chemistry, Wrocław University, Joliot-Curie St. 14, 50-383 Wrocław, Poland; 5 Department of Botany, The Jan Kochanowski University in Kielce, Świętokrzyska St. 15, 25-406 Kielce, Poland; 6 GEOTOP/UQAM, Department of Earth and Atmospheric Sciences, 201 ave du Président Kennedy, Montréal, H2X 3Y7 Canada

**Keywords:** Bioindicators, Polish National Park, Atmospheric pollution, Principal components analysis, Air quality, Multi-parameter analysis

## Abstract

SO_2_, NO_x_, and metals (including Cd, Cu, Pb, Zn, Mn, Mg, Fe) present in airborne particulate matter are a major threat to preserving good air quality. The complicated pathways and transformation processes that can change their physical/chemical state in the atmosphere renders identifying their origin extremely difficult. With the objective of alleviating this difficulty, we identified and characterized potential local and regional sources of atmospheric pollutants using bioindicators (*Hypogymnia physodes*) from the Świętokrzyski National Park (SE Poland): 20 lichen samples were collected during winter (February; heating period) and summer (June; vegetative period) seasons and analyzed for metal contents and free radicals concentrations. Our results indicate that the highest gaseous pollutant levels were observed during the heating season, along roads (NO_2_) and at the highest elevation (SO_2_). The semiquinone/phenoxyl radical concentrations correlated during the heating season with the atmospheric SO_2_: ln (free radicals concentrations) = 0.025 SO_2atmosphere_ + 39.11. For Mn/Fe ≥ 2, the electron paramagnetic resonance (EPR) spectra presented a hyperfine splitting. Results showed that since 1994 metal concentrations increased for Cd, Mn, and Mg, Fe remained somewhat constant for Zn and Cu but slightly decreased for Pb, in agreement with the phasing out of lead in gasoline. Finally, a principal component analysis (PCA) identified two main factors controlling variability within the analyzed parameters: air pollutants transport over long distances and local fuel combustion by both transport and home heating.

## Introduction

Bioindicators such as mosses, needles, and lichens have now proven their added value when evaluating environmental quality, and especially air quality (e.g., Kłos 2007) by naturally cumulating the impacts of chemical contaminants over time, very often in amounts usually exceeding their physiological needs (Bargagli and Nimis 2002). As reported by Conti and Cecchetti (2001), bioindicators are helpful in identifying and sometimes quantifying inputs from anthropogenic activities. Bioindicators, among which mainly lichens, are very sensitive proxies for atmospheric contamination and so are recording precious information about gaseous contaminants, including SO_2_, NO_2_, O_3_ (Conti and Cecchetti 2001; Garty 2001; Lackovicova et al. 2013; Pescott et al. 2015). This attribute is due to physiological properties such as a lack of cuticle, the absence of protective organs (which could limit the adsorption of toxic substances), and a large exchange surface. This is also why they are capable of absorbing both soluble and insoluble mineral nutrients; they survive by maximizing their uptake of atmospheric aerosols and precipitations while minimizing loss (Gerdol et al. 2002). Limitation of the biomonitoring approach is that it yields a time-averaged value over the lifetime of the bioindicator that can be difficult to assess (Sutton et al. 2004).

EPR (electron paramagnetic resonance) spectroscopy has been widely used to study spin centers, the chemical species with unpaired electrons with spin S = 1/2, present in biological organisms. These include both radicals produced by different processes and paramagnetic metal ions playing different important roles. Additionally, EPR spectra, characterizing the free radical types and their concentrations, can bring the information about the vitality conditions of the investigated organisms (Laggner et al. 1988; Stegmann et al. 1991; Lisowski et al. 1993).

EPR analysis has already demonstrated that it is a viable tool for monitoring impacts of atmospheric pollutants, which are reflected through variations in concentration of spin centers (e.g., semiquinone radicals produced by oxidation of polyphenols, Fe(III) and Mn(II) ion agglomerates in biopolymers, and dispersed Mn(II) ions) usually present in biological systems (Jezierski et al. 1999). Moreover, previous studies demonstrated that when EPR is applied to bioindicators, such as lichen thallus, the coupled dependence of the g parameter value and the spin concentration of semiquinone radicals gives both qualitative and quantitative information about corresponding atmospheric gaseous pollutants (e.g., Jezierski et al. 1999). Studies were also carried out to verify the hypothesis that relations exist between atmospheric SO_2,_ NO_2_, and metal concentration and free radical concentration in lichen thallus. Conti and Cecchetti (2001) showed that the accumulation of metals in plants, including lichens, depends upon many factors, including (i) availability of metals, (ii) plant characteristics (e.g., species, age, health, and reproduction type), (iii) temperature, (iv) available moisture, and (v) substratum characteristics. While contaminant in lichen is possible through normal uptake and indirect pathways (mist, dew, dry sedimentation, gaseous absorption; Conti and Cecchetti 2001), concentrations of trace elements found in the lichen thallus can be directly correlated with those present in the environment (Conti and Cecchetti 2001 and references within). Metals ions are transported in atmosphere mainly as a compounds aggregated in particulate matter (e.g., PM_10_ or PM_2.5_) or as ions in precipitation (Jickells et al. 1984; Limbeck et al. 2009). With that in mind, our main objective was to evaluate the possibility of creating a useful environmental tool for testing the environmental state (both quantitatively and qualitatively) of a given study area, by correlating the results of the EPR analysis to the metal contents in lichens. The goal of this study was to study whether the presence of atmospheric gaseous contaminants and metals contained in the mineral fraction of aerosols derived from local (roads, home heating) or regional sources (long distance transport from, e.g., industry pollutants) reflect in the composition of bioindicators for a given area. To fulfill this purpose, two analytical periods were selected, one corresponding to the heating season (winter, with expected higher anthropogenic input) and another corresponding to the vegetative season (summer, with no heating and thus an expected lower anthropopressure stress). We also investigated the influence of characteristics such as elevation, road traffic, human presence, by collecting bioindicator samples at different elevations, along roads, in the vicinity of habitations.

## Study area

The Świętokrzyski National Park (ŚNP) is one of the 23 National Parks existing in Poland and is located within the Świętokrzyskie Voivodship in the southeastern part of Poland. The ŚNP is located between the 50°50′22.94′ and 50°58’0.69″N parallels and between 20°48′24.98″ and 21°4′38.53″ E meridians. The park is surrounded by cities: Kielce on the West, Bodzentyn, Skarżysko-Kamienna, Starachowice on the North and Ostrowiec Świętokrzyski on the East. The major sources of contaminant emissions are located in the north and east parts of the voivodship. The ŚNP was established in 1950 and is located within the central part of the Świętokrzyskie Mountains. The highest peaks are Łysica (612 m a.s.l.), Agata (608 m a.s.l.), and Łysa Góra (595 m a.s.l.). The Świętokrzyski National Park covers 7626.45 ha (among which 23% are strictly protected) whereas the buffer zone covers 20,786.07 ha. The Świętokrzyski National Park is a typical woodland park where 95% (7212 ha) of its surface is forested (Harabin 2000). While the whole Świętokrzyskie Voivodship is characterized by average annual temperature reaching 7.5–8.0 °C, the Święty Krzyż meteorological station (located in the Łysogóry mountain range) indicates an average annual temperature of 5.7 °C. The warmest month is July with an average temperature of 15.7 °C, whereas the coldest one is January with a mean temperature reaching − 4.6 °C (Report 2006). The highest annual average sum of precipitation was observed in Święty Krzyż at 823 mm (Report 2006). In the apical part of the Łysogóry mountain range, the annual sum of precipitation is 800–850 mm annually, whereas the lowest part of the National Park (Bodzentyn) receives 550–600 mm annually (Olszewski et al. 2000). The maximum sum of precipitation is usually observed in July (~ 100 mm) and the minimum in February (~ 30 mm; Report 2006). On the investigated area, wind direction prevails from the south (45.02%). Wind direction from west represents 40.16% and those from southeast and east represent 8.25 and 4.80%, respectively (Olszewski et al. 1994).

### Sampling point localization

**Fig. 1:**
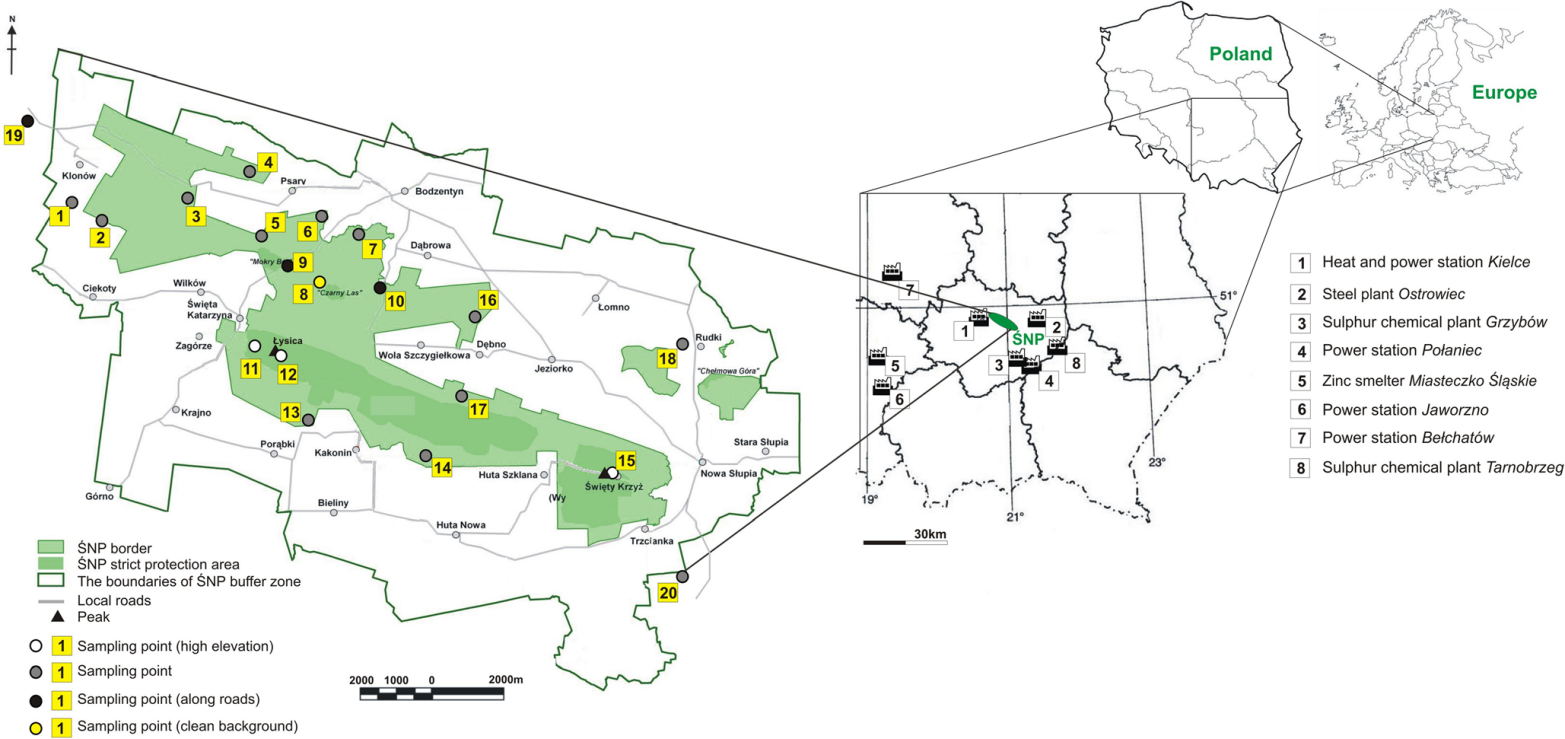
Sampling point map

The detailed map of the sampling sites selected is presented in Fig. 1.

Twenty sampling sites were selected at altitudes varying between 284 and 597 m a.s.l. (Table 1). Characteristics and location of sampling locations included three points along main roads (9, 10, and 19) and three points on the highest elevation of the ŚNP (11, 12, and 15). Sampling point 8 represented the local natural background. The remaining sites (1–7, 13–14, 16–18, and 20) reflected influences from various sources (e.g., industry, home heating).

**Table 1:** Atmospheric contaminants, metal, and free radical concentrations in *Hypogymnia physodes* lichens collected in the Świętokrzyski National Park in 2013. For metals: Zn, Pb, Cd, Cu, Mg, Mn standard deviations (1 sigma) based on CRM analysis was calculated

Sampling Point No.	Altitude [m. a.s.l]	Winter											Summer										
		SO_2_ [μg·m^−3^]	NO_2_ [μg·m^−3^]	g parameter	Free radicals concentration [spins·g^−1^]	Zn [ppm]	Pb [ppm]	Cd [ppm]	Cu [ppm]	Mg [ppm]	Fe [ppm]	Mn [ppm]	SO_2_ [μg·m^−3^]	NO_2_ [μg·m^−3^]	g parameter	Free radical concentration [spins·g^−1^]	Zn [ppm]	Pb [ppm]	Cd [ppm]	Cu [ppm]	Mg [ppm]	Fe[ppm]	Mn [ppm]
1	315	3.5	7.3	2.0037	8.37·10^16^	75.5 ± 3.6	16.1 ± 2.6	1.54 ± 0.2	4.86 ± 0.2	519 ± 24.4	235	583 ± 19.8	0.2	1.4	2.0048	1.28·10^17^	104.0 ± 5.0	13.4 ± 2.2	1.21 ± 0.1	4.50 ± 0.2	516 ± 24.2	533	371 ± 12,6
2	310	5.3	6.3	2.0036	8.35·10^16^	72.9 ± 3.5	13.8 ± 2.2	1.07 ± 0.1	5.96 ± 0.2	368 ± 17.3	348	394 ± 13.4	0.3	1.5	2.0048	1.75·10^17^	109.9 ± 5.3	29.8 ± 4.9	2.07 ± 0.2	11.18 ± 0.4	725 ± 34.1	460	581 ± 19.7
3	327	n.d.	7.9	2.0036	4.42·10^16^	123.0 ± 5..9	16.8 ± 2.7	1.28 ± 0.1	8.46 ± 0.3	508 ± 23.9	685	348 ± 11.8	0.6	1.8	2.0048	5.11·10^16^	134.0 ± 6.4	21.2 ± 3.5	1.87 ± 0.2	13.08 ± 0.5	830 ± 39.0	1020	429 ± 14.6
4	433	10.5	6.0	2.0036	8.24·10^16^	82.1 ± 3.9	11.3 ± 1.8	0.71 ± 0.1	6.87 ± 0.2	476 ± 22.4	567	577 ± 19.6	0.7	2.5	2.0047	1.69·10^17^	113.0 ± 5.4	15.5 ± 2.5	1.04 ± 0.1	6.06 ± 0.2	564 ± 26.5	521	708 ± 24.1
5	317	0.8	6.4	2.0036	1.15·10^17^	75.9 ± 3.7	20.9 ± 3.4	0.86 ± 0.1	5.74 ± 0.2	526 ± 24.7	576	367 ± 12.5	0.9	1.8	2.0047	1.58·10^17^	97.4 ± 4.7	13.0 ± 2.1	1.04 ± 0.1	5.68 ± 0.2	520 ± 24.4	341	259 ± 8.8
6	367	6.3	10.6	2.0035	5.89·10^16^	119.0 ± 5.7	16.3 ± 2.7	1.33 ± 0.1	6.99 ± 0.2	715 ± 33.6	698	1070 ± 36.4	0.6	2.3	2.0048	6.50·10^16^	101.0 ± 4.9	12.7 ± 2.1	0.83 ± 0.1	6.38 ± 0.2	592 ± 27.8	543	500 ± 17.0
7	408	6.4	9.3	2.0037	1.14·10^17^	87.5 ± 4.2	26.5 ± 4.3	2.59 ± 0.3	6.02 ± 0.2	405 ± 19.0	330	398 ± 13.5	0.4	2.0	2.0046	7.59·10^16^	97.0 ± 4.7	18.2 ± 3.0	0.74 ± 0.1	6.01 ± 0.2	661 ± 31.1	460	444 ± 15.1
8	321	n.d.	n.d.	2.0045	8.11·10^16^	98.0 ± 4.7	13.7 ± 2.2	1.30 ± 0.1	4.57 ± 0.2	797 ± 37.4	316	503 ± 17.1	n.d.	1.6	2.0047	1.25·10^17^	122.0 ± 5.9	11.8 ± 1.9	1.92 ± 0.2	4.28 ± 0.2	1120 ± 52.6	247	488 ± 16.6
9	318	1.6	13.2	2.0036	8.79·10^16^	105.0 ± 5.7	16.4 ± 2.7	1.44 ± 0.2	6.71 ± 0.2	507 ± 23.8	473	232 ± 7.9	0.3	9.4	2.0048	1.91·10^17^	114.0 ± 5.5	21.4 ± 3.5	1.09 ± 0.1	7.41 ± 0.3	567 ± 26.6	696	147 ± 5.0
10	295	6.7	9.2	2.0035	8.30·10^16^	69.1 ± 3.3	7.5 ± 1.2	0.54 ± 0.1	6.59 ± 0.2	1140 ± 53.6	768	448 ± 15.2	1.0	4.8	2.0037	9.22·10^16^	86.0 ± 4.1	12.5 ± 2.0	0.94 ± 0.1	10.12 ± 0.4	1383 ± 65.0	515	309 ± 10.5
11	484	12.1	8.6	2.0036	5.39·10^16^	146.0 ± 7.0	24.6 ± 4.0	1.98 ± 0.2	8.40 ± 0.3	565 ± 26.5	349	320 ± 10.9	1.4	2.6	2.0048	1.06·10^17^	160.9 ± 7.7	40.8 ± 6.6	1.31 ± 0.1	15.44 ± 0.5	811 ± 38.1	576	279 ± 9.5
12	597	10.4	5.9	2.0037	7.81·10^16^	135.0 ± 6.5	41.6 ± 6.8	1.63 ± 0.2	9.61 ± 0.2	366 ± 17.2	721	112 ± 3.8	1.0	1.8	2.0047	4.25·10^16^	152.0 ± 7.3	56.8 ± 9.3	1.35 ± 0.1	13.00 ± 0.5	460 ± 21.6	907	98 ± 3.3
13	428	2.8	5.8	2.0037	1.21·10^17^	102.0 ± 4.9	13.9 ± 2.3	0.93 ± 0.1	6.83 ± 0.2	520 ± 24.4	505	355 ± 12.1	0.6	2.3	2.0038	1.11·10^17^	136.0 ± 6.5	21.5 ± 3.5	1.76 ± 0.2	6.73 ± 0.2	613 ± 28.8	437	392 ± 13.3
14	382	3.4	7.1	2.0035	1.09·10^17^	104.0 ± 5.0	24.1 ± 3.9	2.51 ± 0.3	6.34 ± 0.4	623 ± 29.3	284	548 ± 18.6	1.1	2.1	2.0037	1.48·10^17^	87.0 ± 4.2	21.0 ± 3.4	2.25 ± 0.2	6.62 ± 0.2	706 ± 33.2	284	577 ± 19.6
15	591	13.8	6.1	2.0036	1.15·10^17^	141.0 ± 6.8	24.4 ± 4.0	1.04 ± 0.1	10.90 ± 0.2	414 ± 19.4	762	168 ± 5.7	1.9	2.9	2.0036	1.25·10^17^	176.7 ± 8.5	57.1 ± 9.3	2.03 ± 0.2	14.45 ± 0.5	843 ± 39.6	689	362 ± 12.3
16	289	4.8	6.1	2.0036	1.61·10^17^	107.0 ± 5.1	12.1 ± 2.0	2.47 ± 0.3	6.46 ± 0.2	595 ± 28.0	520	454 ± 15.4	0.6	1.8	2.0037	1.94·10^17^	107.0 ± 5.1	15.2 ± 2.5	1.10 ± 0.1	5.59 ± 0.2	655 ± 30.8	449	543 ± 18.5
17	378	13.0	6.4	2.0036	1.03·10^17^	92.3 ± 4.4	15.4 ± 2.5	1.16 ± 0.1	6.33 ± 0.2	716 ± 33.6	453	780 ± 26.5	0.6	1.9	2.0037	1.66·10^17^	109.0 ± 5.2	18.7 ± 3.0	2.08 ± 0.2	5.38 ± 0.2	695 ± 32.7	313	388 ± 13.2
18	284	5.7	6.3	2/0036	1.20·10^17^	70.2 ± 3.4	14.9 ± 2.4	0.76 ± 0.1	4.48 ± 0.2	848 ± 39.8	502	1240 ± 42.1	<0.1	0.9	2.0036	1.76·10^17^	77.8 ± 3.7	13.0 ± 2.1	1.08 ± 0.1	4.69 ± 0.2	894 ± 42.0	367	1360 ± 46.2
19	365	8.9	7.6	2.0036	8.42·10^16^	89.9 ± 4.3	19.9 ± 3.2	1.03 ± 0.1	5.62 ± 0.2	722 ± 33.9	563	380 ± 12.9	1.0	5.0	2.0048	5.80·10^16^	117.0 ± 5.6	26.1 ± 4.3	2.18 ± 0.2	6.03 ± 0.2	466 ± 21.9	776	193 ± 6.6
20	378	14.2	6.8	2.0037	9.45·10^16^	99.0 ± 4.8	16.0 ± 2.6	0.65 ± 0.1	7.64 ± 0.3	397 ± 18.7	547	206 ± 7.0	1.3	2.6	2.0047	1.36·10^17^	98.5 ± 4.7	14.1 ± 2.3	1.31 ± 0.1	5.74 ± 0.2	666 ± 31.3	320	428 ± 14.5
Min	284	0.8	5.8	2.0035	4.42·10^16^	69.1	7.5	0.54	4.48	366	235	112	<0.1	0.9	2.0036	4.25·10^16^	77.8	11.8	0.74	4.28	460	247	98
Max	597	14.2	13.2	2.0045	1.61·10^17^	146.0	41.6	2.59	10.90	1140	768	1240	1.9	9.4	2.0048	1.94·10^17^	176.7	57.1	2.25	15.44	1383	1020	1360
Average	379	7.2	7.5	2.0036	9.37·10^16^	99.7	18.3	1.34	6.77	586	510	474	0.8	2.7	2.0043	1.27·10^17^	115.0	22.7	1.46	7.92	714	523	443
Median	366	6.4	6.8	2.0036	8.60·10^16^	98.5	16.2	1.22	6.53	523	513	396	0.6	2.1	2.0047	1.26·10^17^	109.5	18.5	1.31	6.22	664	488	410
Standard deviation	91	4.3	1.9	0.00021	2.70·10^16^	23.4	7.4	0.62	1.61	193	163	282	0.5	1.9	0.00053	5.00·10^16^	25.7	13.7	0.50	3.56	225	207	264

### Samples collection

#### Passive samplers

Atmospheric NO_2_ and SO_2_ concentrations at each sampling site were determined using two passive samplers hanged at 2 m height above the ground. Passive samplers were exposed for a 1-month period (01 Feb—03 Mar 2013 for winter and 11 Jul–16 Aug 2013 for summer). Passive samplers were made of black polythene tubes with an internal diameter of 25 mm and 10 mm depth (adapted from the Amay-Krochmal method for passive samplers). The two sampling screens were made of stainless steel (23 mm diameter, 0.2 mm mesh size, 0.1 mm wire diameter). Before sampling, the two screens were impregnated with an aqueous solution of 10^−2^ M triethanolamine (TEA; C_6_H_15_NO_3_) solution for trapping both NO_2_ and SO_2_. The screens were placed into the passive sampler with a polypropylene fiber windscreen that protected them against dust and wind (Krochmal and Kalina 1997a, b).

#### Lichens

Lichen investigated in this study were from the *Hypogymnia physodes* (L.) Nyl. species, which is a lichenized fungus, classified as a chlorolichen (Biazrov 2012), with Trebouxia phycobiont (Cuna et al. 2007). The multifoliose thallus is forming a rosette with inflated lobes (mostly 3–7 cm in diameter; Bystrek 1997; Biazrov 2012). The smooth and shining lobes are green-gray or gray on the top cortex, black on the bottom cortex, and light brown on the edges. Lichen samples were collected twice: (i) 1–3 February 2013 (heating/winter season) and (ii) 11–13 June 2013 (vegetative/summer season). For each of the 20 sampling locations, between 1 and 20 g of *Hypogymnia physodes* lichen samples were collected from bark of trunks and twigs of *Abies alba* at a 1–2-m height above the ground, depending on material availability. Lichens were taken from trees using a plastic knife. Lichen samples were then placed into paper envelopes that were taken back to the laboratory.

## Methods

### Determination of atmospheric NO_2_ and SO_2_ concentrations

Analyses of atmospheric NO_2_ and SO_2_ were carried out by employees of the Voivodship Inspectorate for Environmental Protection (VIfEP) in Kraków, delegacy in Nowy Sącz. A DIONEX ICS-1100 chemically suppressed ion chromatograph with a CDD-6A conductivity detector was used. Analytical conditions were as follows: dionex analytical columns IONPAC AS9-SC (4 × 250 mm), guard columns IONPAC AG9-SC (4 × 50 mm), and suppressor ASRS-1. A 1.8 mM Na_2_CO_3_/1.7 mM NaHCO_3_ solution was used as the eluent at a flow of 1.0 ml·min^−1^ for a 20-μl sample size. Temperatures of both the column and the detector were set at 40 °C. NO_2_ and SO_2_ concentrations were calculated following the method described in by VIfEP Krochmal and Kalina (1997a, b) using the equation

$$ x=\frac{1.44\cdot {10}^5\cdot m}{P\cdot t} $$

where *x* is the concentration of NO_2_ or SO_2_ in μg·m^−3^ at 1.013 × 10^5^ Pa and 20 °C, *m* the mass of NO_2−_ or SO_4_^2−^ found in the sampler in μg, *P* an empirical coefficient defined as the mass of NO_2−_ or SO_4_^2−^ in μg determined in the sampler after a 24-h exposure in air containing 100 μg of NO_2_ or SO_2_ per 1m^3^, and *t* the exposure period in minutes.

### Lichen analysis

Each lichen sample was carefully washed three times with deionized water and then air-dried at room temperature, in order to eliminate any outer source of contamination such as pollens, dust particles, dead insects, spider web (Jezierski et al. 1999; Gałuszka 2005).

#### Metal analysis

For metal analysis (Zn, Pb, Cd, Cu, Mg, Fe, and Mn concentrations), lichen samples were digested for 0.5 h in a mixture of 2 mL of deionized water and 8 mL of HNO_3_ using a CEM MARS 5 mineralizer. Metal concentrations were measured on a Thermo Scientific iCAP 7400 ICP-OES DUO using CTA-OTL-1 as standard. Each analysis was tripled and the resulting average standard deviation were as follows: Cd = ± 0.12 μg·g^−1^, Cu = ± 0.50 μg·g^−1^, Zn = ± 2.4 μg·g^−1^, Pb = ± 0.80 μg·g^−1^, Mn = ± 14 μg·g^−1^, Fe and Mg = ± 210 μg·g^−1^. Analyses were carried out according to our internal procedure PBW 11 issue 4 (28.07.^2014^).

#### EPR analysis

Before EPR analysis, a 2-mm section of the edge of each dried lichen sample was cut using a clean ceramic put into a glass tube. The EPR spectra were then recorded, at room temperature, using a Bruker Elexsys E500 spectrometer operating at X-band, equipped with a NMR teslameter (ER 036TM) and a frequency meter. For quantitative measurements, a standard of known spin concentration (Leonardite humic acid, prepared and distributed by IHSS) was placed in the first cavity of the double resonator and the sample in the second one. After tuning, the measurement parameters were set, the EPR spectra were recorded separately for each cavity (i.e., one for the standard and one for the sample. The analysis of the radical quantity (by double integration within the same magnetic field region), in the studied sample and in the Leonardite standard, was carried out using the Bruker WinEPR Processing software, version V2.22Rev.12.

### Statistical analysis

Principal component analysis (PCA) was carried out on our sample dataset, as conventional correlation analysis did not show statistically significant correlations. All calculations were performed using software Statistica 12 (StatSoft Inc. 2014). Metal concentrations in lichens was expected to be controlled by several factors/processes both natural and anthropogenic, and we hoped that PCA could help in discriminating among them. According to Manly (1998), the PCA method involves limited number of useful variables and describes relations among them. All the standardized parameters that we determined (height, free radicals concentration, Zn, Pb, Cd, Cu, Mg, Fe, Mn, and atmospheric NO_2_ and SO_2_) presented normal or close-to-normal distributions (tested using the Shapiro-Wilk test), guarantying representativeness of data and reliable results in the PCA (Drever 1997; Manly 1998). During the PCA transformation factors and its quota (determining using the scree test), factor loadings (determining using normalized varimax rotation), and factor scores were calculated for winter and summer seasons (Cattell 1966; Johnson 1978; Drever 1997).

Scores obtained for factors 1 (explaining 38% (winter) and 43% (summer) of the variations) and 2 (explaining 19% (winter) and 14% (summer) of the variations) are presented in Fig. 3.

## Results

For this study, the concentration of atmospheric SO_2_ and NO_2_ were measured. Additionally, in lichen samples were determined the concentration of the following metals: Zn, Pb, Cd, Cu, Mg, Fe, and Mn. Moreover, the EPR analysis carried out on lichen samples unable to characterize the type of free radicals present and their respective concentrations. Semiquinone or phenoxyl free radicals, discriminated by the g parameter, were detected in each sample analyzed. All the results obtained in this study are gathered and presented in Table 1, separately for winter and summer campaign.

## Discussion

### Characterization of the environmental conditions based on the study of the atmospheric SO_2_ and NO_2_ concentrations

For both the SO_2_ and NO_2_ concentrations, values were higher during the winter compared to the summer season. Fossil fuel combustion is one of the major sources of SO_2_, which would explain why during our study its atmospheric concentrations were almost 10 times higher during the heating season (compared to the vegetative season, defined as the period for which the daily average temperature is higher than 5 °C, Ciężka et al. 2016). A similar situation was observed at the whole voivodship scale, with atmospheric SO_2_ concentrations higher during the heating period of the year (VIfEP website, online measurements), even though a slight decrease was observed between 2010 (15.8 μg·m^−3^) and 2013 (11.0 μg·m^−3^) at Kielce station (Voivodship Inspectorate for Environmental Protection). In the Świętokrzyskie Mountains, the vegetative period lasts 185 days annually (Bróż 2015). Still, the measured SO_2_ concentrations did not exceed the hourly health protection threshold of 350 μg·m^−3^ or its corresponding daily threshold of 125 μg·m^−3^, as well as the plant protection guidelines set at 20 μg·m^−3^ (Dz. U z 2012 r., poz. 1031). The decrease observed between 2010 and 2013 might result from the reduction of yearly SO_2_ emissions within the whole voivodship during the same period, from 29.1 thousand tons in 2008 to 14.1 in 2012 (VIfEP report 2013).

SO_2_ concentrations recorded during our study in the Świętokrzyski National Park were slightly lower than those observed in its closest vicinity during the same period. SO_2_ concentrations in the ŚNP ranged from 0.8 to 14.2 μg·m^−3^ in winter and from < 0.1 to 1.9 μg·m^−3^ in summer (Table 1). On the other hand, the three VIfEP monitoring stations (Kielce - Jagiellońska St., Połaniec and Małogoszcz) recorded SO_2_ values of 24.3, 13.1, and 12.8 μg·m^−3^ during winter, respectively. During summer, SO_2_ concentrations were slightly lower for these monitoring stations: 3.2, 3.8, and 8.1 μg·m^−3^, respectively (VIfEP website, online measurements).

For both seasons, the highest SO_2_ concentrations were measured at sampling points (nos. 11, 12, and 15) located at the highest elevations. SO_2_ concentration also showed a rough positive correlation with elevation (in m a.s.l.) with R^2^ of 0.35 and of 0.43 (*p* = 0.05) for winter and summer seasons, respectively. This hypothesizes that this atmospheric SO_2_ is controlled by long-distance transport rather than originating from local sources, such as factories or power stations located in the ŚNP vicinity (e.g., 3. Sulfur chemical plant Grzybów, 4. Power station Połaniec, 8. Sulfur chemical plant Tarnobrzeg in Fig. 1). As the prevailing wind direction in the region originates from the South (Olszewski et al. 2000), these industries are likely the most probable vectors for the high SO_2_ concentration observed at sampling point no. 20 during the two sampling campaigns.

During winter, higher SO_2_ concentrations were also observed in some sampling points (nos. 4, 6, 7, 17, and 18) influenced by emissions from fuel combustion of home furnaces households located in their closest vicinity. Moreover, higher SO_2_ values recorded during both winter and summer campaigns at point nos. 10 and 19 (and No. 14 but only during summer) might also be linked with domestic heating.

NO_2_ is another gaseous pollutant that needs to be taken into consideration when studying air quality (e.g., Cyrys et al. 2012; Gibson et al. 2013). Polish guidelines established an acceptable NO_2_ annual average concentration of 40 μg·m^−3^ and an hourly average concentration of 200 μg·m^−3^. Between 2008 and 2012, the NO_2_ emissions at the scale of the voivodship slightly increased from 17.6 to 19.6 thousand Mg (VIfEP Report 2013). Despite this, the annual atmospheric NO_2_ concentration in the voivodship displayed a somehow constant level around 25 μg·m^−3^ between 2010 and 2012 (Voivodship Inspectorate for Environmental Protection (VIfEP) 2016), well under the Polish guidelines. During the same period, NO_2_ concentrations in the three monitoring points of the VIfEP (Kielce-Jagiellońska St., Połaniec and Małogoszcz) were higher than those measured within the investigated area. Seasonal variations were observed in the voivodship between 2010 and 2012, with higher average NO_2_ concentrations during winter (38.6, 33.6, and 23.8 μg·m^−3^, respectively; VIfEP website, online measurements) and lower values in summer (22.8, 15.0, and 7.0 μg·m^−3^, respectively). This trend was also observed within the ŚNP area with higher winter NO_2_ concentrations (from 5.8 to 13.2 μg·m^−3^) and lower summer values (from 0.9 to 9.4 μg·m^−3^).

Similarly to SO_2_, atmospheric NO_2_ also presented significant differences among sampling localizations with highest values recorded along roads, e.g., point no. 9 (13.2 μg·m^−3^ for winter and 9.4 μg·m^−3^ for summer), no. 10 (9.2 μg·m^−3^ for winter and 4.8 μg·m^−3^ for summer) for all campaigns, and no. 19 (5.0 μg·m^−3^) during the summer campaign. It could thus be hypothesized that in the SNP area, road traffic might be the dominant source of atmospheric NO_2_. During winter, high NO_2_ concentration were also measured at point nos. 6 (10.6 μg·m^−3^) and 7 (9.3 μg·m^−3^), in the vicinity of households, which may indicate the input of fuels burned in home furnaces.

### Characterization of environmental conditions based on the relationship between gaseous pollutants (SO_2_ and NO_2_) and concentrations of free radicals

**Fig. 2:**
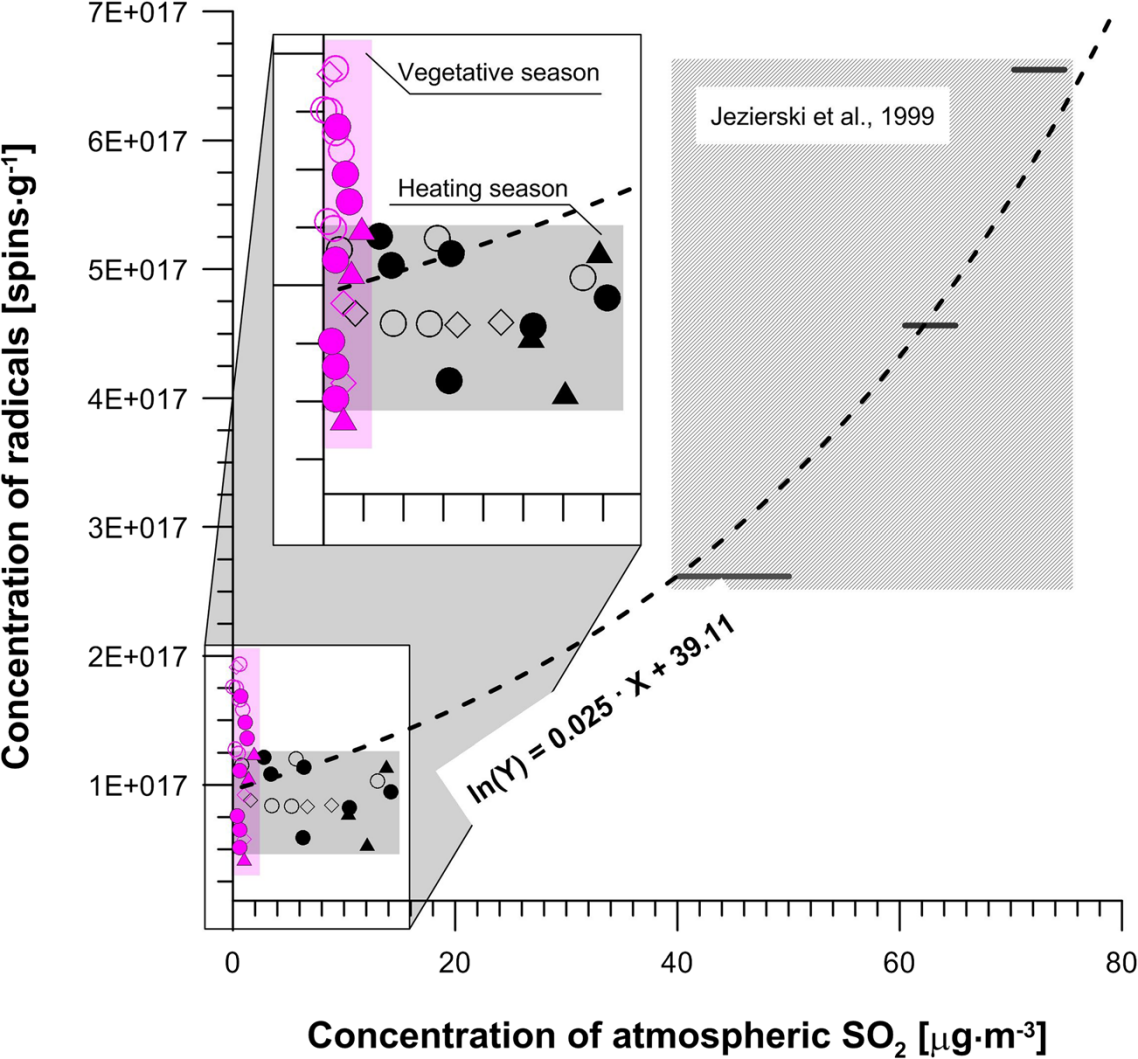
Variations of the atmospheric SO_2_ concentration and the concentration of radicals measured in bioindicators. Results of this study are compared to those reported by Jezierski et al. (1999). Gray lines represent the average concentration of atmospheric SO_2_. The dashed curve is the regression curve calculated for all data, using an exponential function: ln(Y) = 0.025 X + 39.11, r^2^ = 0.95

As previously discussed, lichens are sensitive to various air contaminants, especially sulfur dioxide, nitrogen oxides, metals, ozone, and free-radical forms of oxygen. EPR spectroscopy was used as a sensitive and convenient tool for rapid screening and early monitoring of various air contaminants. Jezierski et al. (1999) postulated that stable radicals generated in long-living lichens are untimely related to air pollution, especially to both SO_2_ and NO_x_ concentrations. Unlike preliminary data reported by Jezierski et al. (1999) for the Karkonosze Mts. study area, our dataset did not exhibit such significant relationship between gaseous pollutants (SO_2_ and NO_2_) and free radical concentration at the Świętokrzyskie Mt. Nevertheless, it should be noted that the study made by Jezierski et al. (1999) (i) took place during the 90s when atmospheric SO_2_ concentrations were up to 10 times higher than those currently observed nowadays and (ii) was based on annual averages compared to monthly ones in our study (Fig. 2, Table 1), and moreover investigated significantly more samples. Still, there is a high probability that concentrations of free radicals are strongly correlated with atmospheric SO_2_ concentrations. However, during the summer season while SO_2_ concentrations yielded a narrow range of values, the concentrations of free radicals showed more variations (Fig. 2). It can be argued that the SO_2_ concentrations we measured were either too low to identify any relationship and/or that SO_2_ was disturbed by secondary atmospheric processes during our study. Another possibility is that the presence of oxides can lead to the formation of ROS (reactive oxygen species; Weissman et al. 2005; Nash 2008; Bhattacharyya et al. 2014; Das and Roychoudhury 2014). The decrease of oxide concentrations in summer would then result in an increase of the free radical concentration. This would be in contrast to the coupled increase of the radical concentration and of SO_2_ concentration obtained by Jezierski et al. (1999). There are two possible mechanisms that would explain it: (i) the scavenging of ROS radicals by polyphenols by providing hydrogen atoms to the ROS radicals, which would lead to an increase of the semiquinone concentration or (ii) an unidentified process leading to the recombination of ROS and semichinone radicals due to electron pairing, which would thus diminish the concentration of semichinones. To explain the trends observed in Jezierski et al. (1999) and our dataset, while this second process needs to be more dominant when concentrations of the nitrogen and sulfur oxides are relatively low (as in our study), the scavenging process of ROS radicals by polyphenols needs to control the ROS budget for higher oxide concentration (as in Jezierski et al. 1999).

**Fig. 3:**
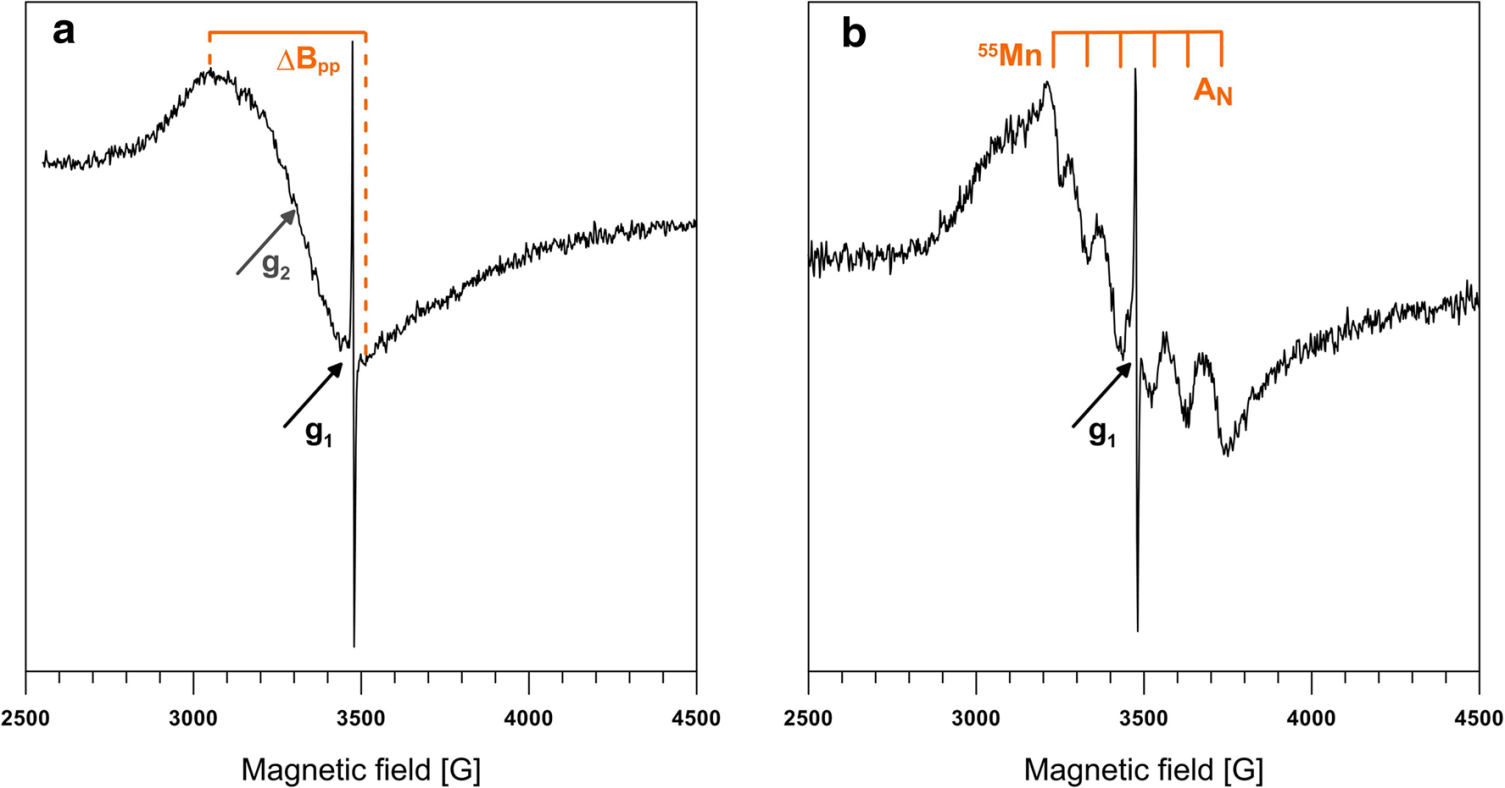
Typical EPR spectra obtained from lichen samples, at 295 K. **a** Sample collected at point 9 during winter. **b** Sample collected at point 18 during summer. g_1_: free-radical; g_2_: high-spin Fe^3+^, A_N:_ hyperfine splitting of Mn^2+^

PCA analysis revealed that the free radical concentration was negatively correlated to the Fe concentration in our lichen samples. We also observe a positive relationship between the SO_2_ and free radicals’ concentrations in the lichen thallus during the winter season when we include results reported by Jezierski et al. (1999) that can be described by the following regression equation (calculated using average values of atmospheric SO_2_ and radicals concentrations from both this study and Jezierski et al. 1999): ln(Y) = 0.025·X + 39.11 (r^2^ = 0.95; Fig. 3), where Y is a free-radical concentration, while X is an atmospheric SO_2_ concentration.

Unfortunately, as isotope compositions of local SO_2_ were not measured during this study, it is not possible to use this equation to relate SO_2_ its corresponding sources in the study area. Nevertheless, if we consider δ^34^S values of SO_4_ from precipitations in the ŚNP (+ 3.8‰, unpublished data), which may then hint at fossil fuel combustion as a major vector of atmospheric sulfur. It may then indirectly hint at the same source (fuel combustion) for atmospheric SO_2_ from which SO_4_ derives by oxidation. Nevertheless, our results show that the analysis of free radical concentration enables to estimate the corresponding atmospheric SO_2_ concentration and ultimately to evaluate the atmospheric pollution level at a given location. Inversely, no correlation between SO_2_ and free radical concentration was observed during the summer season. The absence of correlation may have been caused by (1) low SO_2_ concentrations in the summer, (2) secondary processes that are affecting differently SO_2_ between winter and summer, and (3) the presence of reactive oxygen species (ROS) which will alter SO_2_. Further studies are thus required to explain the absence of correlation during summer.

Based on laboratory experiments where lichen samples were exposed to NO_2_ in a closed chamber (at a concentration of 1% for 24 h), Jezierski et al. (1999) postulated that the presence of iminoxyl radicals was correlated to the level of NO_2_: exposure to higher NO_2_ concentrations (i.e., polluted environments) induced higher concentrations of iminoxyl radicals. Still in their study, the authors observed a weak signal of iminoxyl radicals in two natural (i.e., not exposed to NO_2_) samples of *Umbilicaria polyphylla* lichens. Our results revealed that iminoxyl radicals were detected in none of the analyzed lichen samples, but it has to be noted that atmospheric NO_2_ concentrations in our study were at least 10 times lower than those reported by Jezierski et al. (1999; daily NO_2_ concentrations exceeding 100 μg·m^−3^). Hence, the absence of iminoxyl radicals could result to too low atmospheric NO_2_ concentration in the area investigated. Further investigations are thus required to test the hypothesis that in natural environments, the formation of iminoxyl radicals may be controlled by factors other than atmospheric NO_2_.

### Characterization of environmental conditions based on metal concentrations in lichens

The highest Mn, Pb, and Zn concentrations measured in our *Hypogymnia physodes* samples were higher than those reported by Krawczyk et al. (2004), Białońska and Dayan (2005) and Kłos et al. (2008) for the same species in relatively clean sites, with values of 43, 18, and 55 ppm, respectively. They were also higher than those from samples collected during summer in urban agglomerations reported by Jóźwiak (2012), Kłos et al. (2008), and Parzych et al. (2016b): Mn (37–187 ppm), Pb (28–56 ppm), and Zn (71–166 ppm). However, they were lower for Cu (range of 5.1–21 ppm reported in those studies).

We classified the level of air contamination that our lichen samples underwent, based on their metal concentrations, by applying the scale presented by Bargagli and Nimis (2002). Doing so, sampling point nos. 12 and 15 may be classified as lowly contaminated by Cu, sampling point nos. 11, 12, and 15 moderately contaminated by Pb and highly contaminated by Zn (during both winter and summer). All other samples may be classified as highly (sampling point nos. 3, 6, 8–9, 13–14, and 16) and moderately (sampling point nos. 1–2, 4–5, 7, 10, 17–20) contaminated with Zn (Parzych et al. 2016b). Since the phasing out of Pb in gasoline Zn has been recommended as a reliable tracer of traffic emissions compared to Pb (Oliva and Rautio 2004). Leaching from higher plants is the main source of Mn for epigeic mosses (Faus-Kessler et al. 2001). This leaching originates from high concentrations of Mn contained in tree barks (Hauck and Javkhlan 2009) and is known to be detrimental and a limiting factor to the abundance of epiphytic lichens in coniferous forests (Hauck and Javkhlan 2009). Kabata-Pendias (2001) demonstrated that Mn concentrations of about 400–1000 ppm are harmful for terrestrial plants. Our *Hypogymnia physodes* samples from the Świętokrzyski National Park displayed Mn concentrations that reached values between 112 and 1240 ppm and 98–1360 ppm for winter and summer seasons, respectively. A surplus of Mn is harmful to *Hypogymnia physodes* through activating chlorophyll degradation, inhibiting soredium growth, and causing intracellular Fe deficiency (Hauck and Paul 2005). However, according to the same authors, the adult lichen thalli remain unaffected. Iron is reported as a metal alleviating Mn toxicity symptoms in *Hypogymnia physodes* (Hauck and Paul 2005). *Hypogymnia physodes* in our study had high concentrations of Fe (from 235 to 768 ppm and from 247 to 1020 ppm for winter and summer seasons, respectively; Table 1), significantly higher than the average values observed for plants (5–200 ppm; Markert 1992). This might be related to the local geochemical background, as investigated by Gałuszka (2005).

Highest Cu, Zn, and Pb concentrations were found in lichens collected at the highest elevations sampling point no. 12 (591 m a.s.l.), no. 15 (597 m a.s.l.), and no. 11 (484 m a.s.l.). This is in agreement with the conclusions of Sucharová and Suchara (2004a, b) who showed that they had a positive relationship between elevation and metal concentrations, among which Cu and Zn, in *Pleurozium schreberi* moss samples collected in Příbram in the Czech Republic. Kolon et al. (2010) also presented significantly higher Cu, Fe, Pb, and Zn concentrations in *Pleurozium schreberi* at higher elevations in the Tatra mountains (Poland). This may be explained by the increased contribution of aerial deposits from long-range transport at higher and well-exposed elevations (Kolon et al. 2010). The Kielce monitoring station (VIfEP report 2011, 2013; Voivodship Inspectorate for Environment Protection (VIfEP) 2012a, b, 2016) Pb concentrations in PM_10_ showed a rather stable range of values during 2010–2013 with annual average concentrations of 0.03, 0.045, 0.03, and 0.03 μg·m^−3^ respectively for the 4-year period. The guidelines in Poland for Pb content in PM_10_ is set at 0.5 μg·m^−3^. Cd concentrations in PM_10_ also showed stable average values during 2010–2012 (0.8, 0.9, 0.9 ng·m^−3^, respectively) whereas in 2013, they significantly increased to reach 3 ng m^−3^, but still under the guidelines for Cd in PM_10_ (i.e., 5 ng·m^−3^). Hence, the metals (e.g., Pb and Cd) accumulated in lichens in the study area should correlate to pollutants actually observed in the atmosphere. One can thus wonder how fast the pollutant changes in the atmosphere are reflecting in the lichen thalli (e.g., 1 month, 1 year, or longer time). Time reaction of lichens following SO_2_ changes are actually well documented but migration rate for metals from deposited dust to inbuilt form in the lichen are rather slower and not well constrained. One can also question how high should metal concentration in particulate matter be to be detectable in lichens. This is particularly important for our study area where the reported concentrations for Pb and Cd during the last 4 years are low and still below guidelines (VIfEP report 2011, 2013; Voivodship Inspectorate for Environment Protection (VIfEP) 2012a, b, 2016).

Steinnes and Friedland (2006) and Sarris et al. (2009) showed that large amounts of Cu, Pb, and Zn, among other metals, can be transported over long distances in the atmosphere. Baranowska-Bosiacka et al. (2001) exposed *Hypogymnia physodes* samples to several metal contaminants at increasing concentrations and showed that the thalli preferentially accumulated Cu, Pb, and Zn, while Pb was also found in the cell wall and on the surface of the thalli, and that Cu and Zn were able to penetrate the protoplast. Parzych et al. (2016a, b) reported that *Hypogymnia physodes* among all the lichens studied are the species that contained the highest concentrations of Pb. This may result from its corrugated thalli that give this species a very large contact surface that eases retention of xenobiotics (Kłos 2007).

Metal concentrations had been previously measured in *Hypogymnia physodes* growing on coniferous trees in the Świętokrzyski National Park (Migaszewski et al. 1995; Migaszewski et al. 2001). Comparison with our results for Cd, Cu, Fe, Mn, Mg, Zn, and Pb reveals that between 1994 and 2013 only a decrease in Pb concentrations is noteworthy that is probably related to the phasing out of Pb in gasoline in the 90s. Cd, Mn, Fe, and Mg concentrations, on the other hand, increased during the same period. Finally, Zn and Cu concentrations remained stable in 2013 compared to 1994 (Migaszewski et al. 1995, 2001).

### Characterization of environmental conditions based on the coupled study of EPR spectra and selected metal concentrations (Mn, Fe)

The analysis of the EPR spectra obtained from the powdered lichen samples revealed that the major signals arose from four different centers containing the unpaired electrons (Fig. 3a, b): (I)—a narrow isotropic line associated with a g_1_ parameter between 2.0035 and 2.0047 corresponding to the semiquinone or phenoxyl radicals; (II)—a broad line with a g_2_^eff^ ranging from 2.1 to 2.2 corresponding to high-spin Fe^3 +^ ions coupled by spin-spin interactions; (III)—the signal with g = 2 split into six lines at a distance of about 100 G due to hyperfine interaction between electron spin and manganese (Mn) nuclear spin (*I* = 5/2). These parameters are typical for small concentrations of Mn(II) ions diamagnetically diluted by organic matrix; and (IV)—a broad line with a g_3_ value of about 2, characteristic of agglomerated Mn^2 +^ ions.

The correlation between the intensity of Mn(II) and Fe(III) signals and the ion content reveals that when Mn(II)/Fe(III) > 2 (e.g., samples 1, 6, 18 in winter and samples 8 and 18 in summer), the EPR spectrum is dominated by the signal of agglomerated Mn^2+^ ions and the Mn^2+^ signal splits due to hyperfine interaction (Fig. 3b). When the Mn(II)/Fe(III) ratio is smaller than 2, the spectrum is usually dominated by a strong broad line due to spin-spin coupled Fe(III) ions. However, Mn with a higher degree of oxidation but not detected by EPR analysis at X-band may be present in the sample. In this case, only the signal from Fe(III) is observed. Mn is both an essential micronutrient for most plant organisms involved in several important metabolic processes, especially in photosynthesis and an antioxidant-cofactor enzyme. Deficiency in Mn is dangerous for chloroplasts as it disrupts the photosynthesis process (Millaleo et al. 2010) but excess also appears to be harmful (Mukhopadhyay and Sharma 1991). Availability of Mn mainly depends on the soil pH: assimilation by plants is more difficult in alkaline soils. Excessive Mn concentrations can lead to fluctuations in various processes: enzyme activity, absorption, translocation, and utilization of other mineral elements (e.g., Ca, Mg, Fe, and P) and can initiate oxidative stress (Ducic and Polle 2005; Lei et al. 2007). Threshold of the plant tolerance to a Mn excess is highly dependent on the plant species and cultivars or genotypes within a species (Millaleo et al. 2010). The Mn toxicity is strengthened when other elements, such as Ca, Mg, K, Fe, and Si, are present, even at low concentrations (Abou et al. 2002). For lichen samples collected at sun-exposed areas (glades (samples 3), peaks (samples 12, 15), and roadsides (samples 9, 10, 19)), the broad signal due to Fe(III) was much more distinct. Similar effect had been previously observed for tree needles after a few days of air drying (Lisowski et al. 1993) that the authors assimilated to oxidation of EPR silent Fe(II) ions. The same process accompanies plant aging and affects the oxidation potential in the cell (Lisowski et al. 1993). Finally, our results demonstrated that, in general, a strong EPR Fe(III) signal is accompanied by an absent or weak signal of Mn(II). It had been shown that for some plants, Fe deficiency is identical to a Mn excess and that depression in plant yields can be caused by either a deficiency/excess in Mn or a deficiency in Fe but is cannot be caused by an excess in Fe (Agarwala et al. 1964).

Values of the g parameter (a measure of the modification induced to the unpaired electrons by their environment, e.g., atoms, bonds, solvents) between 2.0035 and 2.0048 are typical of phenoxyls or semichinones derived from phenolic or quinine compounds contained in lichen acids (e.g., usinic or divaricatic acids). Semichinons represent transient forms between hydroquinone and quinones in redox equilibrium processes, while hydroquinone represents its reduced and quinone-oxidized forms (Scott et al. 1998; Pedersen 2002). This equilibrium is characteristic of specific enzymatic processes. During these processes, radicals are able to complex with metal ions thus leading to the lowering of the g value (Witwicki and Jezierska 2011, 2013). This may explain why the g values we measured in our lichen samples were apparently lowered in the winter season compared to the summer season. This may be also caused by greater concentrations of atmospheric NO_x_ and SO_2_ in winter, precursors to the formation of acid rains, as Jezierski et al. (2002) and Witwicki et al. (2008) observed lower g values of the semiquinone radicals at lower pH. Tolerance to sulfur and nitrogen oxides differ greatly among plants. Lichens and mosses are one of the most sensitive organisms to these contaminants and can thus be successfully used as SO_2_ pollution indicators (Varshney et al. 2009). Wellburn et al. (1981) and Irving and Miller (1984) showed that the atmospheric SO_2_ and NO_2_ can lead to an ATP (adenosine triphosphate) deficiency, which is an essential compound for plants and lichens to their growth and repair of secondary damages caused by the air pollution. These studies also showed that the negative impact of SO_2_ is amplified when combined with other air contaminants such as, among others, NO_x_. Thus, variations of the g values in our lichen samples appeared to be season-dependent and may indicate that they may undergo one or both of the following processes: formation of metal-radical complexes and variation of the pH due to environmental pollutions.

### Characterization of environmental conditions based on a PCA

We applied a PCA method to parameters measured during both seasons trying to identify the ones controlling the inter-seasonal variability. The analysis was rendered difficult by the fact that we investigated two different media: metals in lichens and atmospheric contaminants. Results (Table 2) indicate that the PCA was able to correlate 70 and 77% of the observed variables for the winter and summer campaigns, respectively. The remaining 27 and 30% constituted random noise that is interpretable using this technique (Drever 1997; Manly 1998).

**Table 2:** Results of principal component analysis (PCA) for samples collected in the Świętokrzyski National Park in 2013. The bold and italic means statistically significant values.

Variable	Winter			Summer			
	Factor 1	Factor 2	Factor 3	Factor 1	Factor 2	Factor 3	Factor 4
Altitude [m a.s.l.]	***0.85***	− 0.27	− 0.08	***0.79***	− 0.06	− 0.27	0.36
Free radical concentration [spins·g^−1^]	− 0.07	0.34	***− 0.76***	− 0.11	0.02	− 0.03	***− 0.84***
Zn [ppm]	***0.80***	−0.19	0.34	***0.80***	0.10	−0.11	0.39
Pb [ppm]	***0.82***	0.13	0.03	***0.84***	0.06	−0.14	0.38
Cd [ppm]	0.44	***0.74***	0.17	***0.59***	−0.05	0.05	−0.27
Cu [ppm]	***0.79***	− 0.53	0.11	***0.67***	0.12	0.27	0.54
Mg [%]	***− 0.71***	− 0.17	0.22	− 0.01	− 0.07	***0.98***	− 0.02
Fe [%]	0.12	***− 0.82***	0.03	0.21	0.31	−0.14	***0.80***
Mn [%]	***− 0.64***	0.06	0.05	− 0.32	***− 0.69***	0.21	− 0.30
Atmospheric NO_2_ [μg·m^−3^]	− 0.14	0.22	***0.84***	− 0.07	***0.94***	0.05	0.00
Atmospheric SO_2_ [μg·m^−3^]	0.35	***− 0.66***	0.23	***0.77***	0.20	0.16	0.05
Factor contribution [%]	38	19	13	43	14	11	10

**Fig. 4:**
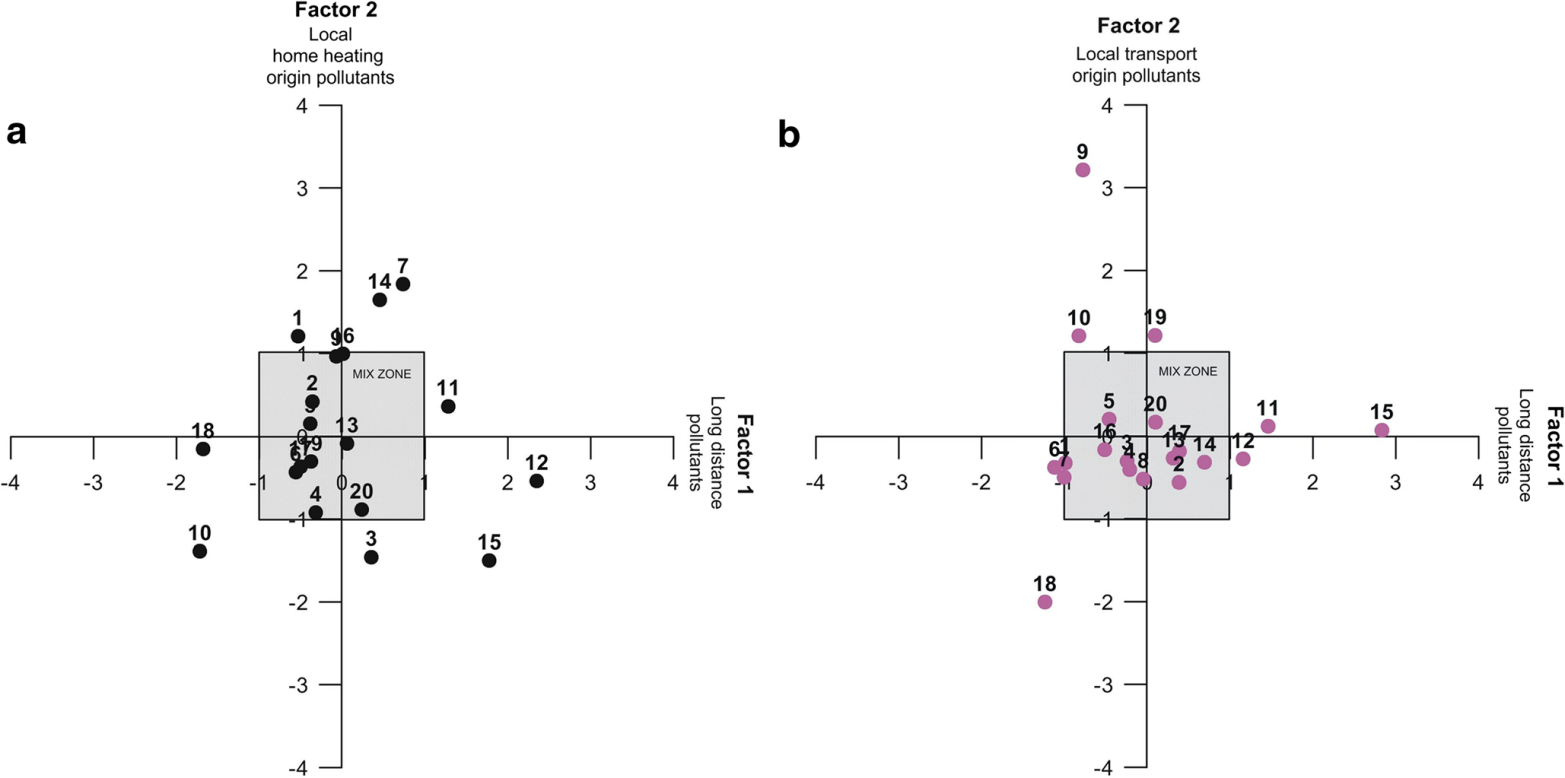
Variations of the principal components analysis factor 1 versus factor 2 for bioindicator samples collected during winter (**a**) and summer seasons (**b**). Samples located within the gray boxes indicate that these were affected indifferently by both factors. Numbers refer to the sample points presented on Fig. 1

During winter, three main factors were identified while in summer, the variability is controlled by at least four different factors (Table 2). During winter, factor 1 explains 38% of observed variations among the studied variables and 42% during summer. For both seasons, positive loadings corresponded to the height, Zn, Pb, and Cu parameters. During winter, negative scores were obtained for macroelements such as Mg and Mn. On the other hand, during summer, Cd and atmospheric SO_2_ concentrations yielded positive scores. Comparing the distribution of factor loading values with the wind directions (S and W) prevailing in the investigated area and the relatively close proximity of Zn–Pb ore deposits and zinc smelter (Fig. 1), factor 1 may be linked to long-distance transport of air contaminants. Comforting this hypothesis, sampling point nos. 11, 12, and 15 that were collected at the highest elevations and that yielded the highest Zn, Cu, and Pb concentrations (Table 1) gave positive scores (> 1.0) for both seasons (Fig. 4a). Surprisingly, during winter season within factor 1, atmospheric SO_2_ was not discriminated with the highest scores obtained at the same highest sampling points (nos. 11, 12 and 15). Nevertheless, during winter, atmospheric SO_2_ was related to factor 2 which accounts for nearly 19% of the total variance. Besides atmospheric SO_2_, Fe had a negative loading and Cd a positive one (Table 2), which hints that factor 2 corresponds to fuel combustion in home furnaces, using pyrites coal or lignite. Fuel combustion could thus be a more important vector for atmospheric SO_2_ than long-distance transport identified in factor 1. Highest factor values were obtained for sampling point nos. 1, 7, 9, 14, and 16 (Fig. 4a) located in places impacted by emissions from home heating. During summer season, factor 2 accounted for almost 14% of the total variance and showed positive loading scores for atmospheric NO_2_ and negative ones for Mn. Highest factor values were reported for sampling point nos. 9, 10, and 19 (Fig. 4b). Based on these results, we can hypothesize that factor 2 probably reflects road traffic as the main process controlling the variance. During summer, tourism significantly increases and thus road traffic emissions (oral information).

Factor 3 accounted for 13% of the total variance during winter and showed positive loading values for atmospheric NO_2_ and negative ones for free radicals in lichen samples. On the other hand, during the summer season, factor 3 and factor 4 accounted respectively for 11 and 10% of the total variance. For factor 3, positive loading value was calculated for Mg. Factor 4 was characterized by positive loading value for Fe and negative ones for free radicals. Unfortunately, these factors (3 and 4) are difficult to interpret.

## Conclusions

The study of atmospheric pollution concentrations combined to metals and EPR characteristics of *Hypogymnia physodes* samples collected in the Świętokrzyski National Park (SE Poland) yielded important information about local environmental conditions. The following conclusions were drawn:

- Higher gaseous pollutants were observed during winter heating season (compared to the summer vegetative season). As expected, highest concentrations of atmospheric NO_2_ were measured along roads while highest concentrations of atmospheric SO_2_ were measured at sampling locations corresponding to the highest elevations.
- Results indicated a relationship between SO_2_ and free radical concentrations (ln(Y) = 0.025·X + 39.11 (r^2^ = 0.95)) during the heating season. It is in agreement with previous studies that showed similar relationships in polluted environments. Still, it has to be noted that this relationship did not exist during the vegetative season in our case, probably resulting from (i) too low SO_2_ concentrations during summer, (ii) additional process(es)/source(s) taking place only during the vegetative season, and (iii) the presence of ROS that can alter the SO_2_ budget.
- The highest Zn, Pb, and Cu concentrations were measured at the highest elevation sampling points, probably indicating contaminant transport over long distances. The high Cd concentrations may be related to vehicle exhausts and TSP aerosol bearing Cd, Mg, Fe, and Mn as macroelements. But the ranges we measured are still compatible with the ranges reported for geochemical background. This could indicate that the lichen samples we collected may not have been impacted by anthropogenic activities when considering metals. Moreover, the high Mn concentrations may be linked with canopy effect.
- The PCA explained 70% of the parameters’ variations for winter and 77% for summer and identified two independent factors controlling their variability: (i) the first linked with long-range transport of pollutants and (ii) the second factor corresponding to local emissions from fuel combustion.
